# Technical efficiency of provincial public healthcare in South Africa

**DOI:** 10.1186/s12962-020-0199-y

**Published:** 2020-01-28

**Authors:** Victor Ngobeni, Marthinus C. Breitenbach, Goodness C. Aye

**Affiliations:** Department of Economics, University of Pretoria, Lynnwood Rd, Hatfield, Pretoria, 0002 South Africa

**Keywords:** Expenditure, Data envelopment analysis, Healthcare, Inefficiency, Technical efficiency, C6-Mathematical programming models, D2-Production and organisations, Fiscal policies and behaviour of economic agents, I1-Health

## Abstract

**Background:**

Forty-nine million people or 83 per cent of the entire population of 59 million rely on the public healthcare system in South Africa. Coupled with a shortage of medical professionals, high migration, inequality and unemployment; healthcare provision is under extreme pressure. Due to negligence by the health professionals, provincial health departments had medical-legal claims estimated at R80 billion in 2017/18. In the same period, provincial health spending accounted for 33 per cent of total provincial expenditure of R570.3 billion or 6 per cent of South Africa’s Gross Domestic Product. Despite this, healthcare outcomes are poor and provinces are inefficient in the use of the allocated funds. This warrants a scientific investigation into the technical efficiency of the public health system.

**Methods:**

The study uses data envelopment analysis (DEA) to assess the technical efficiency of the nine South African provinces in the provision of healthcare. This is achieved by determining, assessing and comparing ways that individual provinces can benchmark their performance against peers to improve efficiency scores. DEA compares firms operating in homogenous conditions in the usage of multiple inputs to produce multiple outputs. Therefore, DEA is ideal for measuring the technical efficiency of provinces in the provision of public healthcare. In DEA methodology, the firms with scores of 100 per cent are technically efficient and those with scores lower than 100 per cent are technically inefficient. This study considers six DEA models using the 2017/18 total health spending and health staff as inputs and the infant mortality rate as an output. The first three models assume the constant returns to scale (CRS) while the last three use the variable return to scale (VRS) both with an input-minimisation objective.

**Results:**

The study found the mean technical efficiency scores ranging from 35.7 to 87.2 per cent between the health models 1 and 6. Therefore, inefficient provinces could improve the use of inputs within a range of 64.3 and 20.8 per cent. The Gauteng province defines the technical efficiency frontiers in all the six models. The second-best performing province is the North West province. Other provinces like KwaZulu-Natal, Limpopo and the Eastern Cape only perform well under the VRS. The other three provinces are inefficient.

**Conclusions:**

Based on the VRS models 4 to 6, the study presents three policy options. Policy option 1 (model 4): the efficiency gains from addressing health expenditure wastage in four inefficient provinces amounts to R17 billion. Policy option 2 (model 5): the potential savings from the same provinces could be obtained from reducing 17,000 health personnel, advisably, in non-core areas. In terms of Policy option 3 (model 6), three inefficient provinces should reduce 6940 health workers while the same provinces, inclusive of KwaZulu-Natal could realise health expenditure savings of R61 million. The potential resource savings from improving the efficiency of the inefficient provinces could be used to refurbish and build more hospitals to alleviate pressure on the public health system. This could also reduce the per capita numbers per public hospital and perhaps their performance as overcrowding is reportedly negatively affecting their performance and health outcomes. The potential savings could also be used to appoint and train medical practitioners, specialists and researchers to reduce the alarming numbers of medical legal claims. Given the existing challenges, South Africa is not ready to implement the National Health Insurance (NHI) Scheme, as it requires additional financial and human resources. Instead, huge improvements in public healthcare provision could be achieved by re-allocating the resources ‘saved’ through efficiency measures by increasing the quality of public healthcare and extending healthcare to more recipients.

## Introduction

According to Statistics South Africa [57], South Africa is a Southern African country with a population of 55.6 million, estimated at 59 million in 2018. Coovadia et al. [15] and Mayosi and Benatar [39] indicate that South Africa is a middle-income country with health outcomes worse than in many low-income countries. This is exacerbated by inadequate human resources to cater for a growing population coupled with a rising number of refugees and economic migrants. As a result, there is growing concern about the state of the public health system, its efficiency and capability to provide sustainable services. The South African health care sector is comprised of public and private segments that are deep-rooted in the past unjust policies of Apartheid, which caused disparities in healthcare access between black and white citizens that there is inequitable access to health services between the poor majority and the rich minority in South Africa. This situation still persists. However, the current divides are generally between the rich and the poor irrespective of race.

The Government of the Republic of South Africa [23] states that, overall, the health sector has 813 hospitals with 133,387 beds for acute health care. The public sector accounts for 49.7 per cent or 404 of these hospitals with 69 per cent of total bed allocation. The private sector comprises 409 hospitals (50.3 per cent) with 31 per cent of the total bed allocation. These numbers clearly point to inequality between the private and public hospitals and may further hint at a shortage of public health infrastructure as these numbers translate into 81,188 people per public hospital with an average of 228 beds per public facility. Mayosi and Benatar [39] add that many state hospitals are in a dire state with much of public healthcare infrastructure run down and dysfunctional due to underfunding, mismanagement, and neglect. This compromises the quality of healthcare and leads to earlier than required patient discharges. The Competition Commission [14] states that, in 2018, the vulnerable and poorly resourced public healthcare facilities served approximately 83 per cent of the population who were largely without medical insurance. The private healthcare facilities served 17 per cent of the population private healthcare insurance. According to the Government of the Republic of South Africa [23], there is a substantial difference in resource availability between the public and private health sectors with more than half of financial and human resources allocated to the private sector.

Marten et al. [36] indicate that, aside from the institutional structure of the healthcare system and inadequate public health infrastructure, another Achilles heel is the absolute and chronic deficit of healthcare workers in the country. Table 1 shows that the existing healthcare workers are unevenly distributed along the health qualification categories and geographical areas. This uneven distribution of staff and skills according to Coovadia et al. [15] has compromised the ability to deliver key programmes, notably for Human Immunodeficiency Virus (HIV), tuberculosis (TB), child health, mental health, and maternal health. Mayosi and Benatar [39] mention that nurses are central to healthcare, especially in rural areas where physicians are reluctant to practice. Table 1 also shows that the nursing personnel accounts for 143,264 or 63 per cent of total health personnel while medical practitioners, specialists and researchers account for 19,988 or 9 per cent. Despite a small component of medical doctors as a proportion of total healthcare workers, 70 per cent are employed in the private sector, implying shortages in the public sector. Moreover, government’s increased investment in the medical profession produced more doctors over the years, but a brain drain has since reversed these gains. South Africa incurs the highest costs for medical doctor education but, in turn, loses returns on investment as doctors migrate to Europe. Thirty per cent of South African doctors have emigrated and 58 per cent are intending to emigrate to Western countries. Community health workers account for 23.7 per cent or 54,180 of health personnel and 4.3 per cent to other health personnel categories.

**Table 1:** Practicing health personnel by sector April 2018. Source: Author’s own Table based on Health Systems Trust [24]

Province	Pupil auxiliary nurses	Student nurses	Enrolled nurses	Nursing assistants	Professional nurses	Medical practitioners	Medical researchers	Medical specialists	Other	Total	% of total
Eastern Cape	356	–	3263	5260	10,993	1903	1	177	6210	28,163	12
Free State	76	–	939	1972	2295	664	5	293	2954	9198	4
Gauteng	823	2902	7694	6518	14,223	3614	13	1929	12,034	49,750	22
KwaZulu-Natal	512	951	9926	5976	17,163	3383	120	808	12,480	51,319	22
Limpopo	64	470	4085	4731	9409	1248	12	60	12,389	32,468	14
Mpumalanga	67	749	1881	1431	5471	1079	1	78	7687	18,444	8
Northern Cape	116	–	237	864	1520	457	2	21	3070	6287	3
North West	49	21	958	2489	4511	934	–	116	6842	15,920	7
Western Cape	225	–	2608	4152	5314	1719	6	1345	5622	20,991	9
Total	2288	5093	31,591	33,393	70,899	15,001	160	4827	64,965	228,217	100

“Other” refers to clinical associates, community health workers, dental practitioners, therapists and specialists, environmental health workers, occupational therapists, pharmacists, physiotherapists, psychologists, radiographers and speech therapists and audiologists. The figures exclude national personnel. Community health workers comprise 83 per cent of the other category

Another major challenge in the healthcare sector is the high cost of providing medical care in South Africa. The Health Systems Trust [25] indicates that this cost is already high and has been increasing rapidly over the last three decades. Moreover, the high public sector salaries and excessive administration costs; duplication of services and inefficiencies are a serious problem. Table 2 shows that the provincial health sector accounted for R186.9 billion of the total provincial budget in 2017/18, which was a 6 per cent contribution to the country’s GDP by all provinces for the same period. The total health spending of R186.9 billion was also equivalent to 33 per cent of total provincial expenditure of R570.3 billion in the same year. The compensation of employees’ budget accounted for 61 per cent of total health expenditure.

**Table 2:** Provincial health contributions to Gross Domestic Product. Sources: Statistics South Africa [56]. National Treasury [42–46, 48–51]

Province	GDP	Health spending	Health spending % GDP
Eastern Cape	247,040,000	22,771,139	9
Free State	154,400,000	9,795,191	6
Gauteng	1,080,800,000	44,132,368	4
KwaZulu-Natal	494,080,000	40,430,163	8
Limpopo	216,160,000	19,522,743	9
Mpumalanga	216,160,000	12,445,693	6
Northern Cape	61,760,000	4,722,157	8
North West	185,280,000	11,420,212	6
Western Cape	432,320,000	21,671,137	5
Total	3,088,000,000	186,910,803	6

*GDP* Gross Domestic Product in R’000 in 2017 terms

Coovadia et al. [15] report that the health sector is also negatively affected by weak political and management leadership. There is insufficient political and management leadership to manage underperformance, especially in the public health sector. This negatively impacts on the efficient administration of the sector and on the provision of quality healthcare services. These challenges have to be resolved urgently, as the Government of the Republic of South Africa [23] reports that South Africa is currently working towards the provision of free quality universal healthcare (UHC) by 2030 which will be publicly funded. Therefore, UHC could require additional financial and human resources prompting the efficient use of existing funding resources. Given the substantial health budgets and their impact on macroeconomic indicators and human development, Verhoeven et al. [60] maintain that there is a general concern about the rapid rise in costs and the trade-offs between efficiency and equity. The Health Systems Trust [25] states that the vision of UHC and the current inefficient delivery of health services are mutually exclusive. As a result, cost containment is an important consideration in the delivery of healthcare as financial and human resources available for healthcare are limited, especially in the public sector where competition for resources is greater, creating a gap between available and required resources for healthcare delivery. Moreover, the National Treasury [47] reports that provincial health departments also face contingent liability risks associated with medical-legal claims due to negligence by health professionals. In 2017/18, this liability was estimated at R80 billion, up 32 per cent from 2016/17. Pay-outs against these claims amounted to R1.5 billion in 2017/18 and are projected to exceed R2 billion in 2018/19.

The Health Systems Trust [25] states that, while it is important to improve the efficiency of existing resources, there is limited available information for efficiency determination. Moreover, scientific economic analysis is not usually used when allocating resources. Given the limited funding of the public healthcare sector and increasing healthcare expenditure requirements, the use of scientific methods to evaluate and compare public healthcare efficiency spending is inevitable and critical in reshaping healthcare policy. The present study fills this gap by using DEA to determine the technical efficiency of provincial healthcare in South Africa. Coelli et al. [13] state that DEA is widely used to measure technical efficiency in the public sector, including in the healthcare sector. Despite this, studies measuring technical efficiency of provincial healthcare sector in South Africa are lacking. Most international studies capturing South Africa compare the national health system with that of other countries without outlining specific provincial health efficiency or inefficiency levels. This is despite the provincial health spending accounting for 33 per cent of provincial annual budgets. A survey of the health sector DEA efficiency assessment literature that was conducted in the current study reflects that the study is the first to assess and quantify in-depth, the technical efficiency of the provincial healthcare sector in South Africa. Therefore, the study generates new knowledge for efficiency benchmarking and improvement. Given the background of the health sector, the rest of the paper is set out as follows: in “Literature review” section, the study conducts a review of literature using DEA to assess healthcare efficiency in the public sector, “Methodology and data” section outlines methodology and data, “Efficiency results” section discusses the efficiency results and “Conclusion” section summarises the findings of the study and details its recommendations.

## Literature review

In terms of DEA literature, in Europe, DEA was used by Campanella et al. [11] to assess the technical efficiency of 50 Italian hospitals. The results revealed an average efficiency score of 77 per cent amongst the DMUs, with a requirement for the inefficient DMUs to reduce their input usage by 23 per cent to achieve efficiency. Lo Storto and Goncharuk [34] employed DEA to measure the technical efficiency of 32 European (EU) countries. The results for model 1 showed that inefficient countries should reduce input usage by 36 and 34 per cent in 2011 and 2014, respectively. The mean efficiency score for model 2 indicated that inefficient DMUs should increase outputs by 68 and 56 per cent for the same years. Asandului et al. [6] also used DEA to analyse the efficiency of healthcare systems of 30 EU countries. The model 1 results respectively showed an average efficiency score of 74 and 75 per cent under the assumptions of CRS and VRS. The model 2 results yielded slightly different average technical efficiency scores of 81 and 77 per cent respectively for the CRS and VRS perspectives. Asandului and Fatulescu [7] used DEA to evaluate the technical efficiency of the healthcare systems of 27 EU countries. The study identified five countries on the efficiency frontier. 14 countries had efficiency scores below 50 per cent, needing to reduce their input usage by more than 50 per cent to be efficient. The remaining countries had efficiency levels of above 50 per cent. Another technical efficiency analytical study in the EU region was conducted by Baciu and Bolezat [9] who assessed the technical efficiency of healthcare of 27 EU countries. The average technical efficiency score of all the DMUs was 60 per cent, showing more room by the inefficient DMUs to reduce the overall input usage by 40 per cent while maintaining the same output levels. Anton [4] measured the technical efficiency of 20 healthcare systems in Eastern and Central Europe. The study obtained an overall mean efficiency score of 98 per cent for life expectancy and 82 per cent for the infant mortality of all the DMUs in the studied healthcare systems. Varabyova and Schreyögg [59] assessed the technical efficiency of healthcare in 31 Organisation for Economic Co-operation and Development (OECD) countries using DEA. The mean efficiency score was 70 per cent, indicating possible input contraction by 30 per cent and possible output expansions by the same proportions. Afonso and St. Aubyn [1] applied DEA to compare the output efficiency of healthcare systems of 30 OECD countries. They ascertained that seven countries were on average technically efficient and inefficient countries could on average increase their output efficiency by 40 per cent. Chowdhury et al. [12] analysed the technical efficiency of 113 acute healthcare hospitals in Canada, a North American country, revealing that most Ontario hospitals were not technically efficient, 65 per cent were subject to decreasing returns to scale (DRS), 10 per cent to increasing return to scale (IRS) and 25 per cent were CRS efficient. Gannon [20] assessed the technical efficiency of 60 Irish hospitals using DEA. The results showed a mean efficiency score of 96 per cent, thereby requiring inefficient DMUs to curb input wastage by 4 per cent.

Other international DEA studies that involved Africa and, in particular, South Africa, are also presented in this section. Alhassan et al. [3] used DEA to assess the technical efficiency of 64 health facilities in Ghana. The results showed an average technical efficiency score of 65 per cent, meaning a reduction in the use of inputs by 35 per cent. DEA was also used by Jarjue et al. [26] to determine the technical efficiency of 41 secondary healthcare centres in the Gambia. The mean efficiency score was 65 per cent, meaning that inefficient DMUs could still increase output by 35 per cent. 10 per cent of the DMUs were scale efficient while 90 per cent were scale inefficient with a mean efficiency score of 87 per cent. Kim and Kang [27] applied DEA to analyse the technical efficiency of healthcare systems of 170 countries, including South Africa. The research found that only 17 per cent of the studied countries used inputs efficiently. Asian countries were the most efficient and South Africa was amongst the inefficient countries, with an efficiency score of 84 per cent wasting about 16 per cent of inputs. High and upper middle-income countries had efficiency scores of over 70 per cent and lower-middle income and lower-income countries recorded the average efficiency scores of 67 per cent and 66 per cent respectively. Additional research by Prasetyo and Zuhdi [54] investigated the technical efficiency of healthcare provision in 81 countries, including South Africa. The study observed that 17 countries were efficient in using government expenditure and South Africa had an efficiency score of 94 per cent. DEA was also used by Marschall and Flessa [35] to compute the technical efficiency of 20 healthcare centres in Burkina Faso. The CRS approach found an average mean efficiency score of 91 per cent and the VRS approach yielded a mean efficiency score of 94 per cent; reflecting a potential increase in outputs by 9 and 6 per cent respectively. Akazili et al. [2] applied DEA to calculate the technical efficiency of 89 randomly sampled healthcare centres in Ghana. They found that 65 per cent of healthcare centres were technically inefficient as they used the resources they did not need. They had an average technical efficiency score of 57 per cent, implying that on average, they could reduce input utilisation by 43 per cent without reducing the prevailing output levels.

In another case, DEA was adopted by Benneyan et al. [10] to compare the technical efficiency of healthcare systems of 180 countries. They found that 115 countries, including South Africa were not efficient. Masiye [38] used DEA to evaluate the technical efficiency of 30 hospitals in Zambia. The overall results showed that Zambian hospitals operated at a 67 per cent level of technical efficiency, implying that 33 per cent of input resources were being wasted. Zere et al. [65] adopted an input-minimisation objective to analyse the technical efficiency of 30 district hospitals in Namibia. The CRS results yielded the average efficiency scores ranging between 63 and 74 per cent over the study period. The mean VRS efficiency scores ranged from 67 to 72 per cent. The study also revealed that IRS were a predominant form of scale inefficiency. Kirigia et al. [28] analysed the technical efficiency of 155 public clinics in KwaZulu-Natal in South Africa. They ascertained that only 47 clinics were technically efficient and 108 were not. The inefficient clinics had room to be efficient by reducing inputs and increasing outputs. They could reduce the number of nurses by 417 and general staff by 457. These reductions represented 31 and 32 per cent inefficiency rates. The outputs could be increased by 115,534 antenatal care visits, 1010 baby deliveries, 179,075 child health care visits, 5702 dental care visits, 121,658 family planning visits, 56,068 psychiatry visits, 34,270 sexually transmitted infections related care visits and 34,270 TB related visits. Lastly, Kirigia et al. [29] assessed the technical efficiency of 55 provincial hospitals in KwaZulu-Natal using DEA. The average technical efficiency score was 90.6 per cent. 22 DMUs were inefficient needing to reduce inputs as follows, 117 doctors, 295 administration staff, 2709 nurses, 835 general staff, 1191 provisioning staff, 61 paramedics, 58 technicians, 38 other staff members and 1752 beds.

In terms of the 21 health sector studies reviewed in this paper, only five related to South Africa. Three of the studies were comparative cross-country efficiency benchmarking studies than relative provincial efficiency analytical studies. These studies used different variables to those applied in the present study. The other two studies compared the efficiency of clinics and hospitals in one of the nine South African province, however, they did not use expenditure as a variable of analysis. The current study focuses on the efficiency of all the provinces, it is specific and explicit in terms of which provinces should be prioritised for reforms, therefore, enabling benchmarking and improvement.

## Methodology and data

In this paper, we follow the DEA approach developed by Farrell in 1957 and enhanced in 1978 by Charnes, Cooper and Rhodes (also called the CCR model) to convert the fractional linear efficiency estimates into linear mathematical efficiency programmes under the CRS. We also use the VRS approach reported by Gavurova et al. [22] to have been developed in 1984 by Banker, Charnes and Cooper to transform the CCR model to allow for consideration of scale efficiency analysis. This is called the Banker, Charnes and Cooper (BCC) model. The terminology “envelopment” in DEA refers to the ability of the efficiency production frontier to tightly enclose the production technology (input and output variables). Cooper et al. [16] and McWilliams et al. [40] state that DEA was developed in a microeconomic setting and applied to firms to convert inputs into outputs. However, in efficiency determination, the term “firm” is often replaced by the more encompassing DMU. DEA is an appropriate method of computing and analysing the efficiency of public sector institutions as they employ multiple inputs to produce multiple outputs. DEA estimated frontiers do not make any assumptions upfront or specify the functional form related to the production technology. Aristovnik [5] and Martić et al. [37] state that there are input-minimisation and output-maximisation DEA orientation models. The former determines the quantity of inputs that could be curtailed without reducing the prevailing level of outputs to make the DMUs efficient and the latter expands outputs of the DMUs until a combination of inputs and outputs reach the production possibility frontier while holding the levels of inputs constant. However, the selection of each orientation is dependent on the objectives of a particular study.

According to Taylor and Harris [58], DEA is a comparative efficiency measurement tool that evaluates the efficiency of homogeneous DMUs operating in similar environmental conditions and where there is no known relationship between the conversion of inputs and outputs. Wang and Alvi [61] report that DEA only uses the information used in a particular study to determine efficiency and does not take into consideration other factors that are exogenous to the study. DEA measures the distance or derivatives of production functions to determine the extent of DMU’s efficiency deviation from the optimal position. It classifies the DMUs into extremely efficient performers versus inefficient performers. In terms of the DEA methodology, the current study uses both the CCR and the BCC models to test for stability, variability and robustness of the obtained efficiency results. These models are described in the following paragraphs.

Under the CCR model, suppose there are $M$ different number of inputs and $P$ different number of outputs for $N$ DMUs. These quantities are represented by column vectors ${\text{x}}_{ij}$ ($i = \, 1, \, 2, \, 3, \, \ldots {\text{M}},$ $j = 1,\;2,\;3 \, \ldots {\text{N}}$) and ${\text{q}}_{rj}$ ($r = 1, \, 2, \, 3, \, \ldots {\text{P}},$ $j = 1,\,2,\,3 \, \ldots {\text{N}}$) The $M \times N$ input matrix, $X$ and $P \times N$ output matrix, $Q$ represents the production technology for all the N number of DMUs. For each DMU, the ratio of all the output variables over all the input variables is represented by ${\text{u}^{\prime}}\text{q}_{rj} /{\text{v}^{\prime}}{\text{x}}_{i}$. Where ${\text{u}} = P \times 1$ vector output weights and ${\text{v}} = M \times 1$ vector input weights. The optimal weights or the efficiency estimates are obtained by solving a mathematical problem. In the context of the CRS, an efficient DMU operates at most productive scale size (MPSS) or technically optimal production scale (TOPS). Hence, the optimal weights or efficiency estimates are obtained by solving a mathematical problem that is reflected in Eq. 1.

$${\text{Tops}} = \max_{{{\text{u}},{\text{v}}}} ({\text{u}^{\prime}}{\text{q}}_{rj} /{\text{v}^{\prime}}{\text{x}}_{ij} )$$

$$St.$$

1 $${\text{u}^{\prime}}{\text{q}}_{rj} /{\text{v}^{\prime}}{\text{x}}_{ij} \le 1$$

$${\text{u}},{\text{v}} \ge 0$$

Equation 1 shows the original linear programme, called the primal. It aims to maximise the efficiency score, which is represented by the ratio of all the weights of outputs to inputs, subject to the efficiency score not exceeding 1, with all inputs and outputs being positive. Equation 1, has an infinite number of solutions, if (u, v) is a solution, so is αv, αv. To avoid this, one can impose a constraint ${\text{v}^{\prime}}{\text{x}}_{ij} = 1,$ which produces Eq. 2.

$$\max_{{{\text{u}},{\text{v}}}} ({\text{u}^{\prime}}{\text{q}}_{rj} )$$

$$St.$$

2 $${\text{v}^{\prime}}{\text{x}}_{ij} = 1$$

$${\text{u}^{\prime}}{\text{q}}_{rj} - {\text{v}^{\prime}}{\text{x}}_{ij} \le 0$$

$${\text{u}},{\text{v}} \ge 0$$

An equivalent envelopment problem can be developed for the problem in Eq. 2, using duality in linear programming. The dual for $\max_{{{\text{u}},{\text{v}}}} ({\text{u}^{\prime}}{\text{q}}_{rj} )$ is $min\theta , \lambda \theta$. The value of $\theta$ is the efficiency score; it satisfies the condition $\theta \le 1$; it is the scalar measure. Lauro et al. [32] report that λ is an $N \times 1$ vector of all constants representing intensity variables indicating necessary combinations of efficient entities or reference units (peers) for every inefficient DMU, it limits the efficiency of each DMU to be greater than 1. This results in Eq. 3, which represents the CCR-CRS model with an input minimisation orientation.

$$Min\theta , \lambda \theta$$

$$St.$$

3 $$- {\text{q}}_{rj} + Q\lambda \ge 0$$

$$\theta {\text{x}}_{i} - X\lambda \ge 0$$

$$\lambda \ge 0.$$

Avkiran [8] states that the CRS postulates no significant relationship between DMU’s operational size and their efficiency. That is, under the CRS assumption, the large DMUs are deemed to attain the same levels of efficiency as small DMUs in transforming inputs to outputs. Therefore, the CRS assumption implies that the size of a DMU is not relevant when assessing technical efficiency. However, in most cases, DMUs have varying sizes and this becomes a factor when determining their efficiency. As a result, Gavurova et al. [22] mention that in 1984, the CCR formulation was generalised to allow for the VRS. Lavado and Domingo [31] argued that, in practice, there is plethora of factors such as financial constraints that may result in the DMUs not operating at optimal scale. Aristovnik [5] adds that, if one cannot assume the existence of the CRS, then a VRS type of DEA is an appropriate choice for computing efficiency. Gannon [20] advises that the VRS should be used if it is likely that the size of a DMU will have a bearing on efficiency. As such, Yawe [63] cautions that the use of the CRS specification when the DMUs are not operating at an optimal scale results in a measure of technical efficiency which is confounded by scale effects. The solution is to use the VRS as it permits for the calculation of scale inefficiency. The CRS linear programming problem can be modified to account for the VRS by adding the convexity constraint: $N1^{\prime}\lambda = 1$ to Eq. 3, where $N1$ is an $N \times 1$ vector of ones to formulate Eq. 4. Equation 4 represents the BBC-VRS model with an input-minimisation orientation. Therefore, Eqs. 1 to 3 represent the CRS models while Eqs. 4 to 5 represents the VRS models.

$$Min\theta , \lambda \theta$$

$$St.$$

4 $$- {\text{q}}_{rj} + Q\lambda \ge 0$$

$$\theta {\text{x}}_{ij} {-}X\lambda \ge 0$$

$$N1^{\prime}\lambda = 1$$

$$\lambda \ge 0$$

Lauro et al. [32] and Yuan and Shan [64] report that the CCR and the BCC models only differ in the manner the latter includes convexity constraints. Since the current model considers the VRS, the restriction $\sum\nolimits_{i = 1}^{n} {\lambda n = 1}$ is introduced. Ramírez Hassan [55] cautions that, if this restriction is not there, it would imply the application of the CRS model. The same analogy applies to all the inefficient provinces in the sample. That is, the slacks and the radial movements are calculated for all inefficient provinces using Eq. 5. The BCC is adept to calculate pure technical efficiency and inefficiency and when applied with the CCR model, it also measures scale inefficiency. Where, $\sum\nolimits_{i = 1}^{I} {\lambda I = 1}$, a DMU is on a CRS frontier, if $\sum\nolimits_{i = 1}^{I} {\lambda I < 1}$, the DMU is located on the IRS frontier and if $\sum\nolimits_{i = 1}^{I} {\lambda I > 1}$, there is DRS. Given that this study has adopted both the CCR and the VRS with an input-minimisation orientation. The DEA models used in this study also consider the slack movements for the inefficient DMUs. As a result, the models account for the slacks in Eq. 5.

$${\text{Min}} \theta , \lambda j,S_{r}^{ + } ,S_{i}^{ - }$$

$$\theta - \varepsilon \left[ {\mathop \sum \limits_{i = 1}^{M} S_{i}^{ - } + \mathop \sum \limits_{r = 1}^{P} S_{i}^{ + } } \right]$$

5 $$\theta {\text{x}}_{i0} - \mathop \sum \limits_{j = 1 }^{N} {\text{x}}_{ij} \lambda j - S_{i}^{ - } = 0,\quad \mathop \sum \limits_{j = 1 }^{N} {\text{x}}_{ij} \lambda j = X\lambda$$

$$\theta {\text{q}}_{r0} = \mathop \sum \limits_{j = 1 }^{N} {\text{q}}_{rj} \lambda j - S_{r}^{ + } = 0,\quad \mathop \sum \limits_{j = 1 }^{N} {\text{q}}_{rj} \lambda j = Q\lambda$$

$$\mathop \sum \limits_{j = 1 }^{N} \lambda j = 1$$

$$\lambda j,S_{r}^{ + } ,S_{i} ^{ - } > 0$$

Coelli et al. [13] define slacks as input excesses and output shortfalls that are required over and above the initial radial movements to push DMUs to efficiency levels. Both the slack and radial movements are characterised only with the inefficient DMUs. The radial movements are initial input contractions or output expansions that are required for a firm to become efficient. $S_{i} ^{ + }$ and $S_{i} ^{ - }$ in Eq. 5 are the output and input slacks respectively to be calculated with $\theta ,$ and $\lambda n$. $\varepsilon$, is the non-Archimedean constant. Gavurova et al. [22] hint that if the slack variables of a DMU are not equal to zero and the technical efficiency score is lower than one, it is necessary to perform a non-radial shift that is expressed by the slack variables to achieve technical efficiency. In Eq. 5, the slack variables determine the optimum level of inputs that DMUs would have to utilise and the outputs that they would have to produce to become efficient, provided that these DMUs are inefficient. Therefore, the slacks depict the under-produced outputs or overused inputs.

Given the specified model, it is clear that this paper uses the CCR and BCC models with input-minimisation objectives to analyse technical efficiency of provincial healthcare in South Africa for the 2017/18 financial year. This is relevant for this study as the technical efficiency of provincial healthcare considers total health spending and total health staff as inputs. The DMUs have control over these variables, especially on expenditure as opposed to heath outputs. The selected output variable for this study is the IMR. A longitudinal approach was not adopted since the composition of provincial health expenditure remains the same throughout. Table 3 summarises the efficiency analytical data. The literature review presented in this paper shows that total health spending, health staff and the IMRs are commonly used variables to analyse health sector efficiency. Therefore, this paper considers various health production technologies with a maximum three-variables. Data for the IMR and total health staff are actual figures obtained from the audited annual reports of provinces for 2017/18. Data for total health spending is obtained from the National Treasury’s 2017/18 provincial revenue and expenditure data. There are high variations reflected by high standard deviations from the mean with respect to health expenditure across all the provinces. The minimum expenditure of R4.7 billion was recorded in the Northern Cape, with Gauteng spending the sample maximum of R44.1 billion. There were also major variations in the number of total health staff per province.

**Table 3:** Input and output variables. Sources: Eastern Cape Department of Health [17], Free State Department of Health [18], Gauteng Department of Health [21], KwaZulu-Natal Department of Health [30], Limpopo Department of Health [33], Mpumalanga Department of Health [41], Northern Cape Department of Health [52], North West Department of Health [53], Western Cape Government Health [62]. National Treasury [42–46, 48–51]

Provinces	Health inputs		Health output
	x1 (THE)	x2 (THS)	y1 (IMR)
Eastern Cape	22,771,139	40,424	14
Free State	9,795,191	17,301	11.8
Gauteng	44,132,368	66,124	10.1
KwaZulu-Natal	40,430,163	68,125	12.4
Limpopo	19,522,743	33,848	12.4
Mpumalanga	12,445,693	20,421	9.7
Northern Cape	4,722,157	6924	11.6
North West	11,420,212	17,536	8.1
Western Cape	21,671,137	31,549	9.3
Observations	9	9	9
Mean	20,767,867	33,584	11
Minimum	4,722,157	6924	8
Maximum	44,132,368	68,125	14
Median	19,522,743	31,549	12
Standard deviation	12,794,907	20,302	2

THE is the actual total health expenditure or spending measured in R’000

THS is the total number of people or workers employed in the health sector

IMR refers to the number of deaths per 1000 live births of children under 1 year of age

**Fig. 1:**
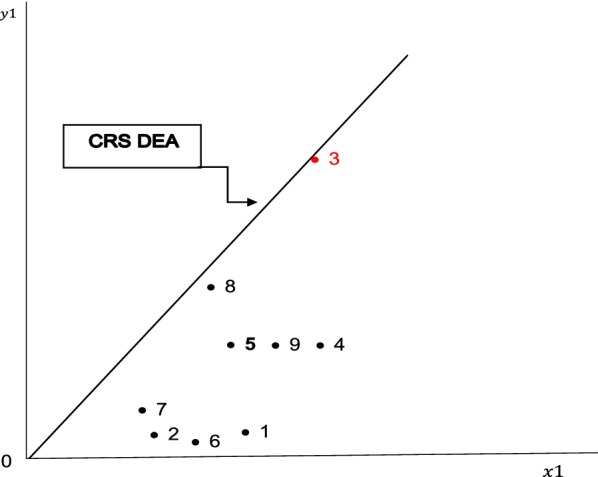
Health model 1 DEA efficiency frontier (Source: Author’s graph based DEAP version 2.1 efficiency results)

**Fig. 2:**
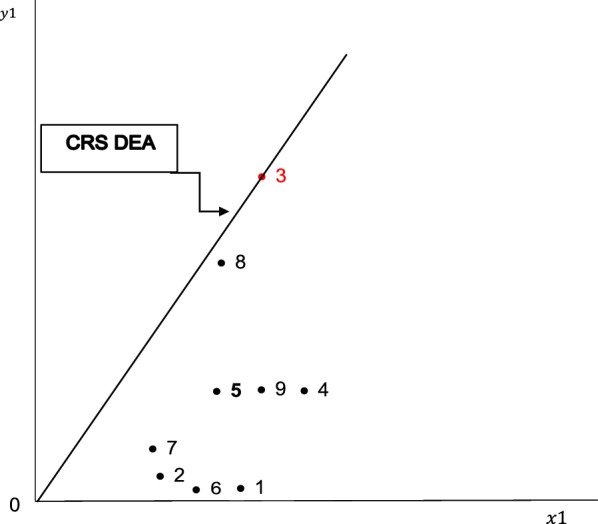
Health model 2 DEA efficiency frontier (Source: Author’s graph based DEAP version 2.1 efficiency results)

**Fig. 3:**
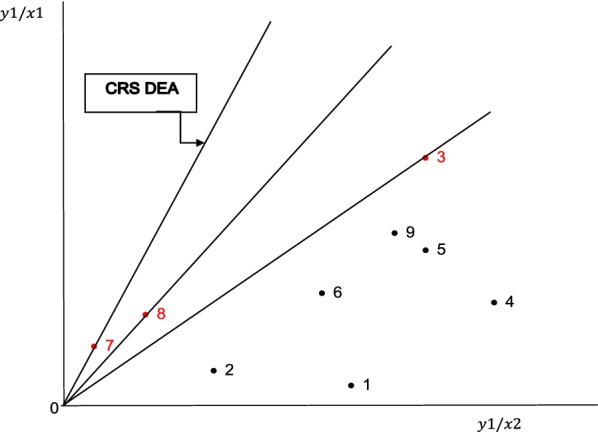
Health model 3 DEA efficiency frontier (Source: Author’s graph based DEAP Version 2.1 efficiency results)

**Fig. 4:**
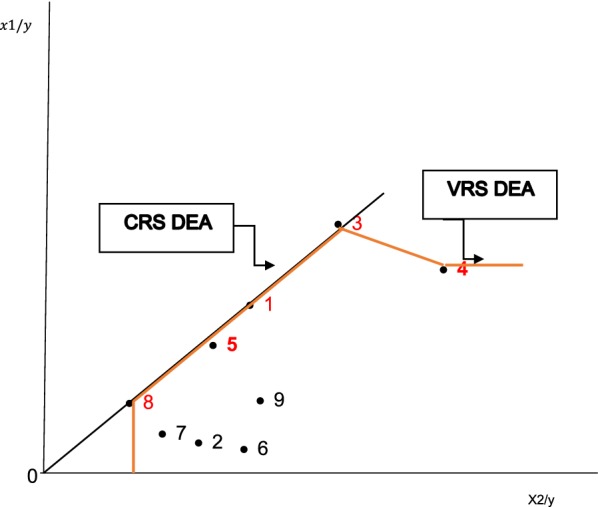
Health model 4 DEA efficiency frontier (Source: Author’s graph based DEAP version 2.1 efficiency results)

**Fig. 5:**
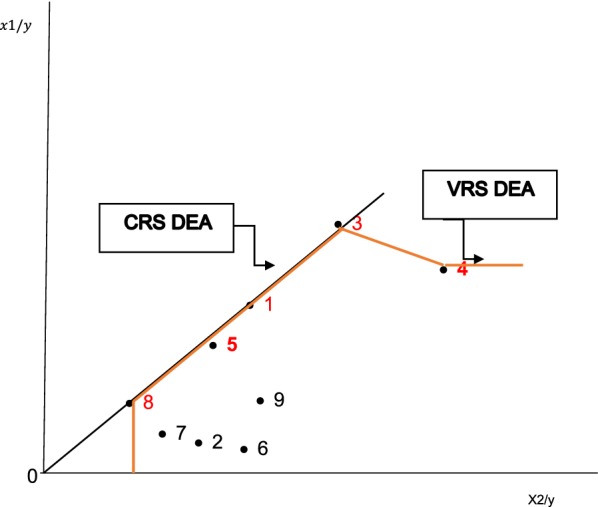
Health model 5 DEA efficiency frontier (Source: Author’s graph based DEAP version 2.1 efficiency results)

**Fig. 6:**
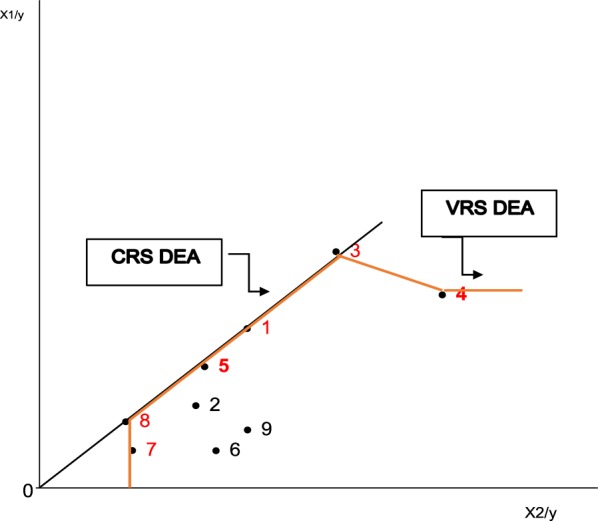
Health model 6 DEA efficiency frontier (Source: Author’s graph based DEAP version 2.1 efficiency results)

The six health models considered in this study are summarised in Table 4, of which three are based on the CRS and three on the VRS. The shapes of the frontiers of these models are illustrated graphically by Figs. 1, 2, 3, 4, 5 and 6 in Appendix.

**Table 4:** Health efficiency DEA models

Models	DEA model	Number of variables	Variable description
Health model 1	CRS	2	Total health expenditure and infant mortality rate
Health model 1	CRS	2	Total health staff and infant mortality rate
Health model 1	CRS	3	Total health expenditure, total health staff and infant mortality rate
Health model 1	VRS	2	Total health expenditure and infant mortality rate
Health model 1	VRS	2	Total health staff and infant mortality rate
Health model 1	VRS	3	Total health expenditure, total health staff and infant mortality rate

*CRS* constant returns to scale and VRS = Variable returns to scale

## Efficiency results

Avrikan [8] reports that the CRS efficiency scores represent technical efficiency. On the other hand, the VRS efficiency scores represent pure technical efficiency. Fried et al. [19] provide intuition that the efficiency results calculated through the VRS are always higher than those calculated using the CRS. This is because the best VRS efficiency frontier is only formed by convex efficient combinations of inputs and outputs. As a result, the BCC model envelops data tighter than the CCR model. Moreover, the VRS is comprehensive as it captures DMUs that are efficient in the CRS and VRS dispensations. Therefore, it contains a smaller number of inefficient DMUs. Table 5 shows the provincial health technical and scale efficiency scores for all the six health models. The health model 1 yielded an average health technical efficiency score of 35.7 per cent, implying that all the inefficient provinces should reduce total health spending by 64.3 per cent while maintaining the same levels of the IMR. Gauteng defined the health efficiency frontier and the North West province was very close to optimality with an efficiency score of 80 per cent. The other seven DMUs had the efficiency scores ranging between 6.4 and 30 per cent, implying inefficiency range of between 70 and 93.6 per cent.

**Table 5:** Health technical and scale efficiency scores. Source: Author’s graph based on DEAP 2.1 efficiency results

Province	Health model 1	Health model 2	Health model 3	Health model 4	Health model 5	Health model 6	Scale efficiency					
Eastern Cape	0.064	0.035	0.063	1000	1000	1000	0.064	DRS	0.035	DRS	0.063	DRS
Free State	0.137	0.137	0.607	0.313	0.313	0.670	0.440	DRS	0.440	DRS	0.906	DRS
Gauteng	1.000	1000	1000	1000	1000	1000	1000	–	1000	–	1000	–
KwaZulu-Natal	0.300	0.300	0.375	1000	1000	1000	0.300	DRS	0.300	DRS	0.375	DRS
Limpopo	0.300	0.300	0.648	1000	1000	1000	0.300	DRS	0.300	DRS	0.648	DRS
Mpumalanga	0.129	0.129	0.504	0.143	0.143	0.542	0.900	IRS	0.900	IRS	0.930	IRS
Northern Cape	0.183	0.183	1000	0.417	0.417	1.000	0.440	DRS	0.440	DRS	1.000	–
North West	0.800	0.800	1000	1000	1000	1000	0.800	IRS	0.800	IRS	1.000	–
Western Cape	0.300	0.300	0.552	0.333	0.333	0.635	0.900	IRS	0.900	IRS	0.870	DRS
Mean	0.357	0.354	0.639	0.690	0.690	0.872	0.572		0.568		0.755	

*CRS* constant returns to scale, *VRS* variable return to scale

The Eastern Cape was the least efficient province with an efficiency score of 6.4 per cent. Mpumalanga records an efficiency score of 12.9 per cent, the Free State 13.7 per cent, the Northern Cape 18.3 per cent, KwaZulu-Natal, Limpopo and the Western Cape had efficiency scores of 30 per cent each. As it relates to health model 2, the mean efficiency score was 35.4 per cent. The efficiency scores for all the DMUs, except for the Eastern Cape were similar to those in health model 1. The Eastern Cape was still the worst performing DMU with an efficiency score of 3.5 per cent. Table 5 reveals an average efficiency score of 63.9 per cent for health model 3 when total health expenditure and total health staff were used together as inputs of the health production frontier while maintaining the same rate of IMR. These variables complemented each other; the use of one without the other decreased the efficiency scores. In this model, Gauteng, the Northern Cape and the North West Provinces were technically efficient. The Free State, Limpopo, Mpumalanga and the Western Cape’s efficiency scores surpassed 50 per cent. KwaZulu-Natal and the Eastern Cape were below 50 per cent with the latter as an extreme inefficient outlier.

When only total health spending was used as an input under the VRS in health model 4, the mean efficiency score was 69 per cent. This was 33.3 per cent higher than the efficiency results that are obtained in health model 1 for the same variable. This implied that the size of provinces matters in determining the technical efficiency of their health spending. Five provinces, the Eastern Cape, Gauteng, KwaZulu-Natal, Limpopo and the North West Provinces were purely technically efficient. This means that four provinces had to reduce total health expenditure inefficiency by 31 per cent. This model also showed that the Eastern Cape, KwaZulu-Natal, North West and Limpopo were largely disadvantaged when scale was disregarded, as they were only efficient under the VRS while they were inefficient under the CRS. The Mpumalanga Province was the least efficient DMU in this scenario with an inefficiency score of 85.7 per cent. It was followed by the Free State, Western Cape and the Northern Cape Provinces with inefficiency rates of 68.7, 66.7 and 58.3 per cent respectively.

The frontier for total health staff in health model 5 was exactly similar to the one for total health spending in health model 4, meaning that the efficiency scores for the DMUs were similar when individually assessing these two variables. As a result, the shapes of the efficiency frontiers of health models 4 and 5 were also similar. This implied that either one of these variables was appropriate to assess the technical efficiency of the health sector under the VRS. The results of the health model 6 also show that when both these input variables were considered under the VRS, the average pure technical efficiency score of the DMUs increased by 23.3 per cent compared to health model 3 results. The health model 6 mean efficiency score was 87.2 per cent, denoting that inefficient DMUs should reduce total health expenditure and total health personnel by 12.8 per cent. Six provinces were purely technically efficient in health model 6. These were the Eastern Cape, Gauteng, KwaZulu-Natal, Limpopo, Northern Cape and the North-West. The inefficient DMUs are the Free State, Mpumalanga and the Western Cape.

Table 5 also shows the scale efficiency scores for the DMUs under consideration. The first column under the heading “scale efficiency” in Table 5 shows the scale efficiency scores derived by dividing the efficiency results of health model 1 by health model 4 results. The second column shows the type of scale efficiency. Therefore, the average scale efficiency score of all the DMUs when total health spending was used as a single variable was 57.2 per cent, depicting high levels of scale inefficiency. The scale inefficient DMUs need to improve scale efficiency by 42.8 per cent. Only Gauteng was CRS scale efficient, providing a benchmark for all the scale inefficient DMUs. The Western Cape, Mpumalanga and the North West Provinces were very close to scale efficiency. They were the only DMUs on the IRS frontier while the other five scale inefficient DMUs were on a DRS curve. The third column in Table 5 indicates an average scale inefficiency score of 56.8 per cent when total health personnel was used as a single input, resulting in 8 DMUs being scale inefficient. The Western Cape, Mpumalanga and the North West Provinces are very close to scale efficiency. They were the only DMUs on IRS frontier while the other five scale inefficient DMUs were on DRS. The last two columns in Table 5 show that when both inputs were employed, the average scale efficiency was 75.5 per cent. Gauteng, Northern Cape and the North West were scale efficient, Mpumalanga was the only DMU on the IRS while the other scale inefficient provinces were on DRS.

Table 6 indicates the radial and slack movements for all the health DEA models. In the health model 1, the mean efficiency score of 35.7 per cent translated into an average inefficiency score of 64.3 per cent. This inefficiency score was equivalent to eight inefficient provinces overusing total health expenditure by R46.4 billion. In other words, total health spending could be reduced by R46.4 billion, while still producing the same output levels. The Eastern Cape could reduce total health spending by R20.6 billion (93.6 per cent inefficiency rate) given the IMR of 14 per cent. The Free State’s and Mpumalanga’s respective inefficient total health spending levels were R6.9 billion (86.3 per cent inefficiency score) and R6.1 billion (87.1 per cent rate of inefficiency) given their current levels of IMR of 11 and 9 per cent. The Northern Cape had overall health spending radial movement of R4.9 billion (81.7 per cent inefficiency score), accounting for 10.6 per cent of the consolidated provincial health spending inefficiency with an IMR of 11 per cent. KwaZulu-Natal, Limpopo, Western Cape and the North West Provinces collectively accounted for 17 per cent or R7.9 billion (associated with their respective 70, 70, 70 and 80 per cent inefficiency rates) of the overall health expenditure inefficiency at the prevailing IMR. In health model 2, all the provinces had a mean health staff inefficiency score of 64.6 per cent, equivalent to 64,400 more health personnel than required. The Eastern Cape had to reduce health workers by 38,600 (96.5 inefficiency weight) while still maintaining the IMR at 14 per cent. The Free State had 6900 (86.3 per cent inefficiency weight) more personnel than it should, Mpumalanga 6100 (87.1 per cent inefficiency score) and the Northern Cape 4900 (81.7 per cent inefficiency score) at the prevailing IMRs. KwaZulu-Natal and Limpopo were each supposed to reduce their total health workers by 2800 (30 per cent efficiency rate) and the Western Cape by 2100 (30 per cent efficiency score). The North West was closest to the efficiency frontier defined by Gauteng, it only had 200 (20 per cent inefficiency rate) more health personnel than required. In terms of health model 3, when total health spending and total health staff were simultaneously applied as inputs, the overall health expenditure mean inefficiency score of 36.1 per cent was equal to R481.8 million total health spending and 49,337 total health personnel in line with an improvement in the efficiency scores from 35 per cent to 63.9 per cent. The Eastern Cape had R397.2 million overall health expenditure inefficiency faced with a daunting task of reducing health sector employees by 37,470 (93.7 per cent inefficiency rate). KwaZulu-Natal was required to curtail total health spending by 42.5 million and total health staff by 2500 in line with a 62.5 per cent inefficiency score to become efficient. All inefficient provinces had to reduce the average technical inefficiency score of 36.1 per cent for both health spending and staff. The Free State used R6.7 million more than it should and could reduce the overall health personnel by 3148 to address its inefficiency rate of 39.3 per cent. The Limpopo province had health expenditure and staff radial movements of R11.6 million and 1407 respectively, associated with its inefficiency rate of 35.2 per cent. The Mpumalanga Province had an inefficiency score of 49.6 per cent; it overused total health spending of R9.9 million and should reduce total health staff by 3469 to become efficient. The Western Cape should reduce health expenditure by R13.9 million and health staff by 1343 (44.8 per cent inefficiency score). The peers for the Eastern Cape, Free State, Limpopo, Mpumalanga and the Western Cape are the North West and the Northern Cape and for KwaZulu-Natal is Gauteng.

**Table 6:** Total health expenditure and health staff radial and slack movements: CRS. Source: Author’s graph based on DEAP 2.1 efficiency results

Provinces	Eastern Cape	Free State	Gauteng	KwaZulu-Natal	Limpopo	Mpumalanga	Northern Cape	North West	Western Cape	Total
Model 1: CRS THE only										
Original input	22,000,000	8,000,000	1,000,000	4,000,000	4,000,000	7,000,000	6,000,000	1,000,000	3,000,000	56,000,000
Input radial movement	(20,600,000)	(6,900,000)	–	(2,800,000)	(2,800,000)	(6,100,000)	(4,900,000)	(2000)	(2,100,000)	(46,400,000)
Input slack movement	–	–	–	–	–	–	–	–	–	–
Input target	1,400,000	1,100,000	1,000,000	1,200,000	1,200,000	900,000	1,100,000	800,000	900,000	9,600,000
Original output	14	11	10	12	12	9	11	8	9	96
Output radial movement	–	–	–	–	–	–	–	–	–	–
Output target	14	11	10	12	12	9	11	8	9	96
DMU peers	3	3	3	3	3	3	3	3	3	
Model 2: CRS THS only										
Original input	40,000	8000	1000	4000	4000	7000	6000	1000	3000	74,000
Input radial movement	(38,600)	(6900)	–	(2800)	(2800)	(6100)	(4900)	(200)	(2100)	(64,400)
Input slack movement	–	–	–	–	–	–	–	–	–	–
Input target	1400	1100	1000	1200	1200	900	1100	800	900	9600
Original output	14	11	10	12	12	9	11	8	9	96
Output radial movement	–	–	–	–	–	–	–	–	–	–
Output target	14	11	10	12	12	9	11	8	9	96
DMU peers	3	3	3	3	3	3	3	3	3	
Model 3: CRS THS and THE										
THS original input	40,000	8000	1000	4000	4000	7000	6000	1000	3000	74,000
THS radial movement	(37,470)	(3148)	–	(2500)	(1407)	(3469)	–	–	(1343)	49,337
THS slack movement	–	–	–	–	–	–	–	–	–	–
THS target	2530	4852	1000	1500	2593	3531	6000	1000	1657	24,663
THE original input	424,000	17,000	66,000	68,000	33,000	20,000	6,000	17,000	31,000	682,000
THE radial movement	(397,181)	(6689)	–	(42,500)	(11,607)	(9912)	–	–	(13,875)	481,764
THE slack movement	–	–	–	–	–	–	–	–	–	–
THE target	26,819	10,311	66,000	25,500	21,393	10,088	6000	17,000	17,125	200,236
Original output	14	11	10	12	12	9	11	8	9	96
Output radial movement	–	–	–	–	–	–	–	–	–	–
Output target	14	11	10	12	12	9	11	8	9	96
DMU peers	7;8	7;8	3	8	7;8	7;8	7	8	7;8	

THE is in R’000

*DMU* decision-making unit, *THE* total health expenditure, *THS* total health staff, *CRS* constant return to scale

Table 7 shows that in terms of the health model 4, only five DMUs were purely technically inefficient with an overall inefficiency score of 31 per cent, translating into total health spending inefficiency of R17 billion. Mpumalanga accounted for R6 billion (85.7 per cent inefficiency score) of this amount, the Free State for R5.5 billion (68.7 per cent inefficiency weight), Northern Cape for R3.5 billion (58.3 per cent inefficiency score) and the Western Cape for R2 billion (66.7 per cent inefficiency score). This overspending could be curtailed while maintaining the same IMRs. The Free State and the Northern Cape should draw lessons from Gauteng and KwaZulu-Natal while Mpumalanga, North West and the Western Cape should learn from Gauteng. The model showed output slacks of 1, 2 and 1 per cent for Mpumalanga, North West and the Western Cape respectively. Given the nature of the IMR, it is not ideal to increase this measure. The same five provinces were inefficient in health model 5. Their mean inefficiency score of 31 per cent was equivalent to 17,000 more health workers than required. The Free State accounted for 32.4 per cent or 5500 of this amount (68.7 per cent inefficiency score), Mpumalanga 35.3 per cent or 6000 (85.7 per cent inefficiency score), Northern Cape 20.6 per cent or 3500 (58.3 per cent) and the Western Cape for 11.8 per cent or 2000 (66.7 per cent inefficiency score) of excess health staff. The model showed the output slacks of 1, 2 and 1 per cent for Mpumalanga, North West and the Western Cape respectively. The Free State’s peers were Gauteng and KwaZulu-Natal and the rest of the DMUs could draw lessons from Gauteng. In terms of health model 6, only three provinces had a mean inefficiency score of 12.8 per cent, equivalent to inefficient total health spending of R26.1 million and 6940 excess health staff. The Northern Cape reached the efficiency point. The Free State had excess staff of 2644 and R5.6 million of health expenditure (33 per cent inefficiency rate), Mpumalanga 3202 and R9.2 million (45.8 per cent inefficiency score) and the Western Cape of 1094 and R11.3 million (36.5 per cent inefficiency weight). The model showed an output slack of 0.7 per cent for Mpumalanga. The Free State and the Western Cape could improve their performance by benchmarking with Limpopo, Northern Cape and North West while Mpumalanga’s peers are the Northern Cape and the North West.

**Table 7:** Total health expenditure and health staff radial and slack movements: VRS. Source: DEAP 2.1 efficiency results

Provinces	Eastern Cape	Free State	Gauteng	KwaZulu-Natal	Limpopo	Mpumalanga	Northern Cape	North West	Western Cape	Total
Model 4: VRS THE only										
Original input	22,000,000	8,000,000	1,000,000	4,000,000	4,000,000	7,000,000	6,000,000	1,000,000	3,000,000	56,000,000
Input radial movement	–	(5,500,000)	–	–	–	(6,000,000)	(3,500,000)	–	(2,000,000)	(17,000,000)
Input slack movement	–	–	–	–	–	–	–	–	–	–
Input target	22,000,000	2,500,000	1,000,000	4,000,000	4,000,000	1,000,000	2,500,000	1,000,000	1,000,000	39,000,000
Original output	14	11	10	12	12	9	11	8	9	96
Output radial movement	–	–	–	–	–	–	–	–	–	–
Output slack movement	–	–	–	–	–	1	–	2	1	4
Output target	14	11	10	12	12	10	11	10	10	
DMU peers	1	4;3	3	4	5	3	4;3	3	3	
Model 5: VRS THS only										
Original input	40,000	8000	1000	4000	4000	7000	6000	1000	3000	74,000
Input radial movement	–	(5500)	–	–	–	(6000)	(3500)	–	(2000)	(17,000)
Input slack movement	–	–	–	–	–	–	–	–	–	–
Input target	40,000	2500	1000	4000	4000	1000	2500	1000	1000	57,000
Original output	14	11	10	12	12	9	11	8	9	96
Output radial movement	–	–	–	–	–	–	–	–	–	–
Output slack movement	–	–	–	–	–	1	–	2	1	4
Output target	14	11	10	12	12	10	11	10	10	100
DMU peers	1	3;4	3	4	4	3	3	3	3	
Model 6: VRS THS and THE										
THS original input	40,000	8000	1000	4000	4000	7000	6000	1000	3000	74,000
THS radial movement	–	(2644)	–	–	–	(3202)	–	–	(1094)	(6940)
THS slack movement	–	–	–	–	–	–	–	–	–	–
THS target	40,000	5356	1000	4000	4000	3798	6000	1000	1906	67,060
THE original input	424,000	17,000	66,000	68,000	33,000	20,000	6000	17,000	31,000	682,000
THE radial movement	–	(5618)	–	–	–	(9153)	–	–	(11,305)	(26,076)
THE slack movement	–	–	–	(35,000)	–	–	–	–	–	(35,000)
THE target	424,000	11,382	66,000	33,000	33,000	10,847	6000	17,000	19,695	620,924
Original output	14	11	10	12	12	9	11	8	9	96
Output radial movement	–	–	–	–	–	–	–	–	–	–
Output slack movement	–	–	–	–	–	0.678	–	–	–	0.678
Output target	14	11	10	12	12	9.7	11	8	9	97
DMU peers	1	5;8;7	3	5	5	7;8	7	8	7;5;8	

*DMU* decision-making unit, *THE* total health expenditure, *THS* total health staff, *VRS* variable returns to scale

## Conclusion

**Fig. 7:**
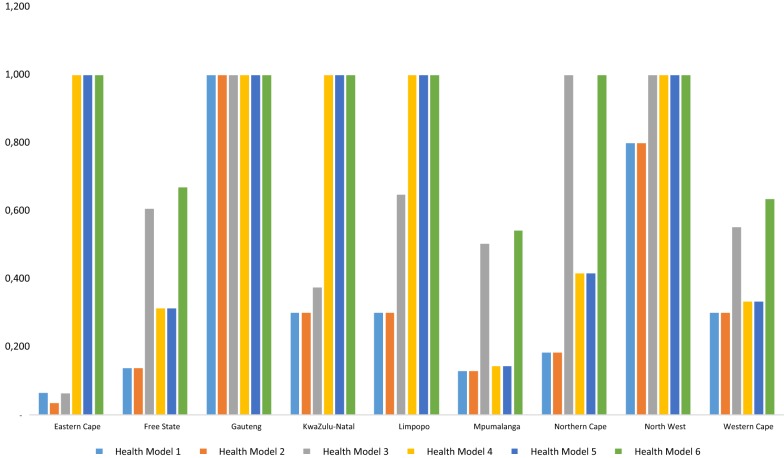
Summary of provincial health technical efficiency model results. Author’s graph based on DEAP 2.1 efficiency results

This study determined the average technical efficiency scores of the six health models. The mean efficiency scores ranged from 35.7 per cent to 87.2 per cent between health model 1 and health model 6. This illustrates that, in line with theoretical postulates, the DEA results under the VRS were higher than under the CRS. The sensitivity analysis was conducted by dropping one input variable in the first two models both under the CRS and the VRS and reintroducing it in the last models under the same assumptions. This exercise showed that the efficiency results improved when the two variables were used simultaneously and decreased when they were used individually under the CRS and the VRS. This further reflects that the health efficiency results are sensitive to the number of inputs used in efficiency analysis. Using a single health input generated lower efficiency results in both assumptions. Figure 7 in Appendix illustrates the individual provincial scores within the broader DMUs’ performance. Gauteng was the best performing DMU defining the efficiency frontier in all the six health models. The second-best performing province was the North West. Other provinces like KwaZulu-Natal, Limpopo and the Eastern Cape only perform well under the VRS. Mpumalanga, Western Cape and the Free State are poorly performing provinces with respect to health sector efficiency.

The efficiency scores of the VRS model (models 4, 5 and 6) are used to formulate the recommendations for this study since this method is comprehensive. The following policy options and recommendations are made.

- Policy option 1 (health model 4): Target minimising total health expenditure in four inefficient provinces, the Free State, Mpumalanga, Northern Cape and the Western Cape. Their collective healthcare expenditure inefficiencies amounted to R17 billion. The Free State should curtail overspending by R5.5 billion and the Northern Cape’s is R3.5 billion. They could draw lessons from Gauteng and KwaZulu-Natal. Mpumalanga was spending R6 billion on healthcare more than it should while the Western Cape should reduce its inefficiency by R2 billion. All the interventions to realise these levels of spending should be implemented while maintaining the prevailing levels of IMRs. These two provinces can benchmark their health operations with Gauteng for pure technical efficiency improvements.
- Policy option 2 (health model 5): Target reducing the overusage of the health staff in the four inefficient provinces. In terms of minimising total health staff, there was overstaffing in the Free State, Mpumalanga, Northern Cape and the Western Cape. Their consolidated health staff inefficiency was 17,000 people. The Free State should reduce health staff complement by 5500 benchmarking with Gauteng and KwaZulu-Natal, Mpumalanga by 7000, Northern Cape by 6000 and the Western Cape by 2000 with all of them benchmarking with Gauteng while maintaining the same rates infant mortality.
- Policy option 3 (health model 6): In cases where the interest of policy makers was to improve health staff and expenditure at the same time, the Free State, Mpumalanga and the Western Cape should respectively reduce staff by 2644, 3202 and 1094 while maintaining the same levels of IMR. The same provinces should be assisted to cut their spending inefficiencies by R26.1 million. The Free State by R5.6 million, Mpumalanga by R9.2 million and the Western Cape by R11.3 million while KwaZulu-Natal should curtail THE by R35 million. The Free State and the Western Cape should learn from Limpopo, the Northern Cape and the North West to improve their efficiency while Mpumalanga should benchmark with the last two provinces.

The potential savings from improving the efficiency of the inefficient provinces could also be used to refurbish and build more hospitals to alleviate the pressure on the public health system. This could also reduce the per capita numbers per public hospital and perhaps their performance as overcrowding is reportedly negatively affecting their performance and health outcomes. Moreover, overcrowded hospitals amid low professional health workers place pressure on the few appointed core health staff complement. This warrants the use of potential savings to appoint more personnel, especially medical practitioners, specialists and researchers while reducing personnel expenditure in non-core areas, as there is a general shortage of healthcare practitioners in South Africa. This implies the improvement of the ratio of practitioners and nursing assistants to attended patients. Moreover, it is essential to retrain health professionals using the realised efficiency gains to reduce the alarming numbers of medical-legal claims. This could also free up additional resources to enhance service delivery.

The study also cautions that, given healthcare personnel and infrastructure challenges, South Africa is not ready to implement the National Health Insurance (NHI) scheme. The scheme requires additional financial and human resources amidst the existing inefficiencies. Instead of taking on the NHI at this juncture, which would be very costly, South Africa can make huge improvements in public healthcare provision by improving efficiency and re-allocating those resources ‘saved’ through efficiency measures, to improving the quality of healthcare and extending healthcare to more recipients. Implementing the NHI without implementing the efficiency measures will set up the health sector for certain failure. In general, provinces should also review the high spending levels on goods and services to ensure value-for-money.

Inefficient provinces should continuously monitor the efficiency of health spending and health personnel and publish their efficiency performance periodically for public scrutiny. This could promote efficient and evidence-based budgeting. In terms of study limitations, this study only determines the inefficiency levels of health spending and health personnel without methodologically explaining the factors resulting in these inefficiencies. As a result, it is difficult to understand why such inefficiencies exist. The study also suffers from a small sample size problem by just analysing nine provinces. However, this limitation is structural given that South Africa has only nine provinces.

## Data Availability

The datasets analysed during the current study are available from the corresponding author on reasonable request. This particularly relates to the DEA model inputs and results. Most of data sources are listed in the reference list.

## Appendix: Health efficiency frontiers

See Figs. 1, 2, 3, 4, 5, 6, 7.
